# Gas permeation through rubbery polymer nano-corrugated membranes

**DOI:** 10.1038/s41598-018-24551-4

**Published:** 2018-04-20

**Authors:** Giuseppe Firpo, Elena Angeli, Patrizia Guida, Roberto Lo Savio, Luca Repetto, Ugo Valbusa

**Affiliations:** Nanomed Labs, Physics Department, University of Genova, Via Dodecaneso, 33, 16146 Genova, Italy

## Abstract

The purpose of this investigation is to fabricate PDMS membranes with reliable surface roughness in order to reduce the surface resistances and to study its impact on the permeation rate. The permeance of CO_2_ through PDMS membranes with rough surfaces at nanoscale is studied and compared with the one of membranes with flat surfaces. At very low thickness, rough membranes have a permeance greater than that of membranes with flat surfaces. The enhancement occurs in a regime where the gas transport is sorption desorption surface rate limited, and cannot be explained by the increase in surface area due to the corrugation. The analysis, introducing a phenomenological model in analogy with electrical flow, indicates that nano-corrugation reduces the surface resistance. To test the model, the permeance of N_2_ is also measured in the same experimental conditions and the influence of surface roughness on permeation rate of CO_2_, He, CH_4_ and N_2_ is studied. The comparison among the gases suggests that the Henry’s coefficient depends on the surface roughness and allows discussing the role of roughness on membrane selectivity.

## Introduction

Rubbery polymer membranes have recently received great attention thanks to their suitability in several areas of application, and, above all, in gas separation^1–5^. A recent paper^6^ has shown that for membranes of small thickness (typically below 250 µm) this class of polymers exhibits an apparent permeability $P$ which, for a given diffusivity *D* and solubility *S*, is lower than the value *P*_*HF*_ = *DS* indicated by Fick’s and Henry’s laws. In particular

1 $${P}={{P}}_{{HF}}\frac{\frac{{L}}{{{L}}_{{C}}}}{1+\frac{{L}}{{{L}}_{{C}}}}$$

where *L* is the membrane thickness and *L*_*C*_ is a characteristic length. Equation (1) accurately reproduces a large number of permeability experimental values of rubbery polymer membranes^5^ having a thickness such that the partitioning process of the gas at the interfaces never reach the equilibrium value^7–9^. The results indicate that for small *L* the measured permeability decreases with *L*, and the length scale *L*_*C*_ determines the transition from Surface Limited Regime (SLR) to Diffusion Limited Regime (DLR). Permeability in SLR is lower than the DLR value *P*_*HF*_. The permeation rate of the membrane is limited by the sorption desorption rate at the surfaces and any further increase can then be achieved by changing the surface kinetics. The surface kinetics limits also the permeance *P*_*L*_ in fact

2 $${P}_{L}=\frac{P}{L}=\frac{{P}_{\mathrm{HF}}}{{L}_{C}(1+\frac{L}{{L}_{C}})}$$

is limited to the value of *P*_*HF*_*/L*_*C*_ as the thickness *L* goes to zero.

The mechanism of the surface sorption desorption limited rate transport is easily described by

3 $${\rm{\Delta }}{p}=\frac{{L}}{{PA}}Q$$

where *A* is the cross-sectional surface area available for permeation, *Q* is the permeation rate and *Δp* the partial pressure difference across the membrane. Equation (3) defines a *membrane resistance R = L/(PA)*, equivalent to the electrical resistance. When the membrane is very thin, feed and permeate surfaces limit the partitioning process, which is not anymore ‘near’ equilibrium. In these conditions a series of feed *R*_*S*_, permeate *R*_*S*_ surface membrane resistances and bulk resistance *R*_*b*_ describes the membrane resistance *R*. *Δp* results

4 $${\rm{\Delta }}{p}=RQ=({{R}}_{{s}}+{{R}}_{b}+{{R}}_{s}){\rm{Q}}=(\frac{{\rho }_{s}}{A}+\frac{L}{{P}_{HF}A}+\frac{{\rho }_{s}}{A})Q$$

As consequence of eqs (1), (3) and (4) ${\rho }_{s}=\frac{{L}_{C}}{2{P}_{HF}}$, ${R}_{b}=\frac{L}{{P}_{HF}A}$.

In terms of resistances, *P*_*L*_ results

5 $${{P}}_{{L}}=\frac{1}{(2{{R}}_{{s}}+{{R}}_{{b}}){{\rm A}}}$$

Since *R*_*b*_ *→* *0* as *L →* *0* from equation (5) appears that *P*_*L*_ is limited by the resistances of the two surfaces *R*_*s*_ and consequently any further increase of permeation rate can be only achieved by their reduction.

At the contrary when *L* >> *L*_*C*_ results *R*_*b*_ >> *R*_*s*_ and the permeance assume the standard value *P*_*L*_ = *P*_*HF*_/*L*.

Since the resistances depend on surfaces, we study the effects on the gas permeation induced by roughening the feed and permeate membrane surfaces.

There are several examples in literature that suggest that surface roughness may increase the permeation rate of membranes. Hirose, Ito and Kamiyama^10^ for instance, studying the relationship between skin layer surface structures of cross-linked aromatic polyamide composite reverse osmosis membranes, observed that membranes, whose skin layer surface structures were rough on the scale of 1 μm, produced higher fluxes. They observed that there is a linear relationship between surface roughness and flux, concluding that the flux increase may be regarded as an enlargement of the effective membrane area. Yave *et al*.^11,12^, studying materials to design membrane for carbon dioxide separation, considered that nano-corrugated surface might contribute to increase the permeability. Gronda, Buechel and Cussler^13^ performing a theoretical model of corrugated membranes, predicted, for small thicknesses, an increase of the flux by a factor *δ* = *A*_*f*_/*A* where *A*_*f*_ is the area of the flattened surface and *A* the cross-sectional surface area of membrane. Goodyer and Bunge^14^ made a more sophisticated mathematical model to explain the mass transfer through membranes with surface roughness. Peters, Lammertink and Wessling^15^ compared flat and micro-patterned membrane having the same volume, observing and predicting a permeability enhancement for the latter. Pisarev *et al*.^16,17^ simulating hydrogen permeation through rough membranes, observed that in SRL, surface roughness affects strongly the dynamics of permeation. They investigated membranes with rough surfaces in different conditions (rough on one side and on both sides) and compared the results with membranes with flat surfaces. In addition, they showed that permeation rate is practically independent on roughness in the Diffusion Limited Regime. Cole, Holter and Pfeifer^18^ studying the adsorption at low coverage on rough surfaces, showed that the Henry’s law coefficient depends on fractal dimensionality of the surface.

All these results indicate the need of further investigations to fully understand the role of surface roughness on the permeation rate. The purpose of this paper is to fabricate membranes with reliable surface roughness at nano scale in order to reduce the surfaces resistances and study its impact on the permeation rate and selectivity. Measurements of membrane permeance at different conditions (rough on one side and on both sides) are carried out and the results are compared with membranes with flat surfaces. The paper considers the case of CO_2_ and N_2_ flowing through PolyDiMethylSiloxane (PDMS) membranes of different thickness and moreover investigates the permeance of CO_2_, He, CH_4_ and N_2_ at different surface roughness. The comparison among the gases allows to discuss the role of roughness on membrane selectivity.

## Method

### Membrane fabrication

**Figure 1:**
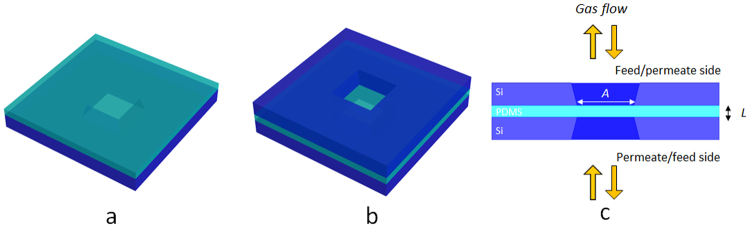
PDMS membrane fabrication. (**a**) PDMS on silicon chip. (**b**) PDMS covered by a second silicon chip. (**c**) Cross section of the PDMS membrane of area *A* and thickness *L*. The membrane is symmetric and can be used from both sides.

The procedure to fabricate the membranes is described in ref.^6^. In the present paper a mask is mounted on the membrane as described in Fig. 1 to guarantee the same exposed area to feed and permeate side. This modification avoids lateral diffusion as observed frequently in composite membranes^19^. Figure 1 shows the device fabrication. A 5 mm × 5 mm commercial square silicon chip with a hole in the center (purchased from Applied NanoStructures, Inc. USA) supports a PDMS film (see Fig. 1a). An identical square silicon chip (the mask) covers the PDMS film (see Fig. 1b). The complete device consisting in a PDMS membrane of thickness *L* having same permeate and feed area *A* is reported in Fig. 1c. The membrane is symmetric and can be used on both sides.

**Figure 2:**
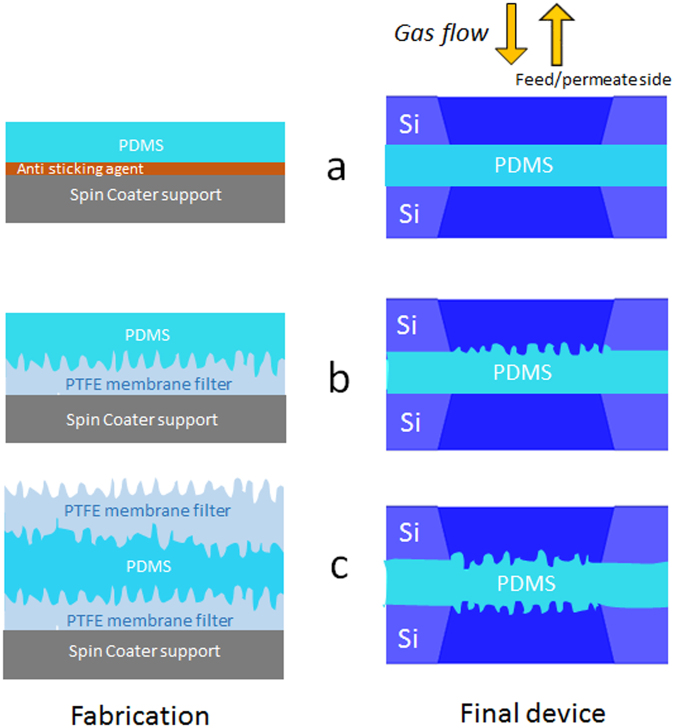
Fabrication and assembly of flat and corrugated membranes. (**a**) Flat FF. (**b**) One side corrugated FC. (**c**) Double side corrugated CC.

Three kinds of PDMS membranes are fabricated: a) a membrane (hereafter reported as FF) with both flat surfaces, b) a membrane (FC) with only one corrugated surface and c) a membrane (CC) with both corrugated surfaces. FF is fabricated as shown in Fig. 2a. The fabrication of corrugated surfaces in FC and CC is reported in Fig. 2b,c. FC membrane is realized (Fig. 2b) by spin coating the non-cured PDMS on a hydrophobic polytetra-fluorethylene (PTFE) filter (0.2 μm pore size, purchased from Sartorium Stedim Biotech GmbH) and subsequently transferred on the silicon chip by the same technique shown in ref.^6^ and schematically reported in Fig. 1. CC membrane is fabricated by the following steps: a) the PDMS film is spin coated on a PTFE filter and non-cured, b) a PTFE filter is placed on the non-cured PDMS film, c) the PDMS film is cured as in ref.^6^, and d) the upper PTFE filter is mechanically removed resulting in a double-corrugated membrane (Fig. 2c). The corrugated surface is a replica of the PTFE filter surface. By changing the filter is possible to change in a reproducible manner the surface roughness. The present paper uses two different membranes with roughness of 86 nm and 220 nm (see Table 1).

**Table 1:** Thickness *L* and cross-sectional surface area *A* of PDMS membranes.

	*R*_*q*_ = 86 nm																*R*_*q*_ = 220 nm
	FC membranes						FF membranes					CC membranes					FC membranes
*L* (μm)	3	7	15	32	43	500	5.3	10	32	70	590	10	15	20	30	1100	30
*A* (μm^2^) × 10^−2^	19	24	42	220	257		23	400	350	235		7	21	18	19		58
*A* (mm^2^)						78.5					78.5					78.5	

Membranes with thicknesses ranging from a few micrometers to about 70 μm are obtained by varying the rotational speed of the spin coater. In order to guarantee a measurable gas flow the dimensions of the cross-sectional surface area are different for different membrane thickness; to maintain a good mechanical stability, if necessary, thinner membranes have smaller area. The membranes with thicknesses in the mm range have been fabricated without spinning, using a Petri’s dishes as support and transferred directly on the copper disk of the gas permeation apparatus following the procedure described in ref.^6^. Table 1 reports the dimensions *L* and *A* for all the membrane tested.

### Characterization

**Figure 3:**
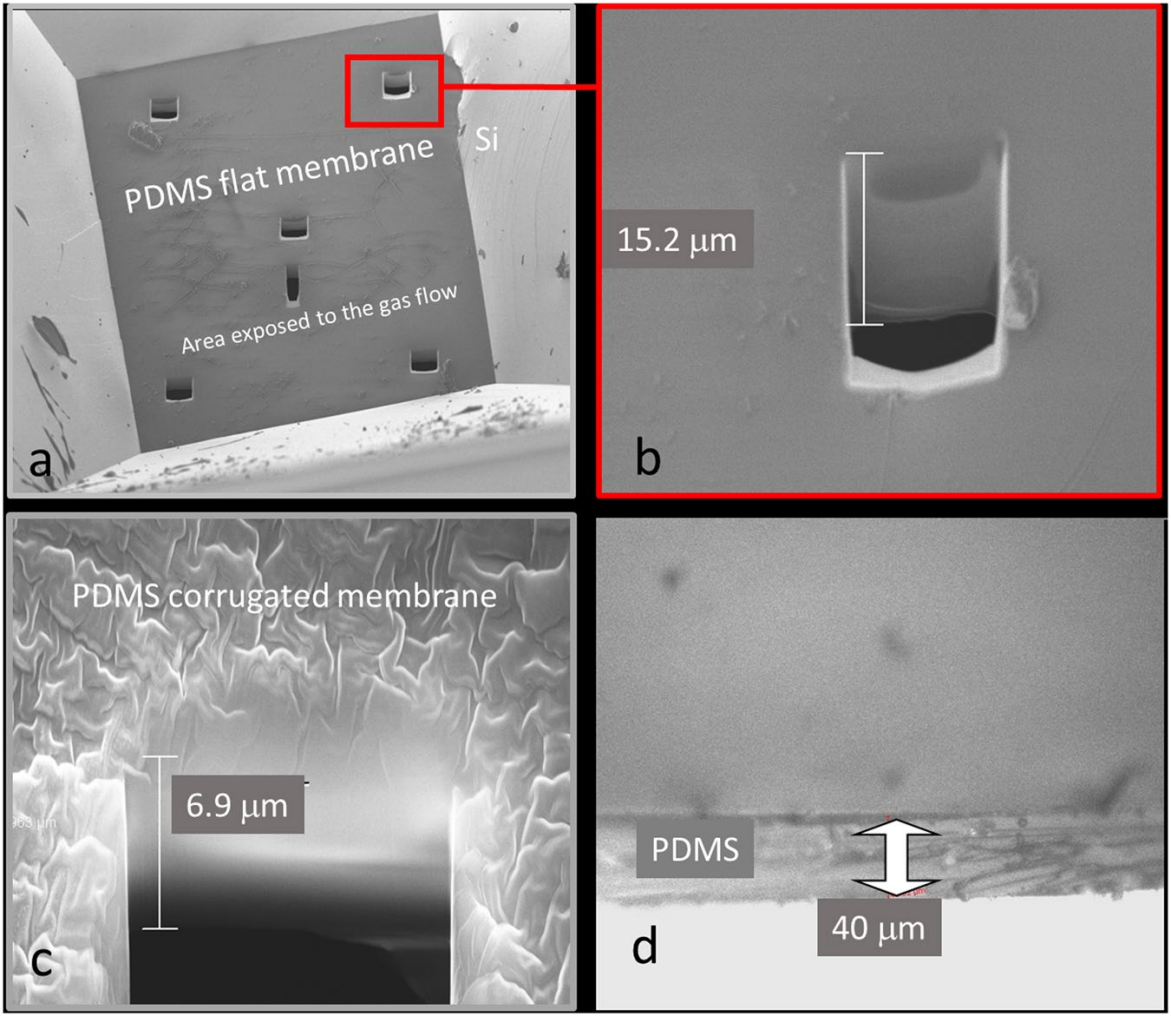
Thickness measurements. (**a**) SEM image of the PDMS membrane with six cross sections obtained by FIB. (**b**) SEM magnification of one of the cross section. (**c**) SEM magnification of a cross section of a thinner membrane with corrugated surface. (**d**) Optical microscope image of a thicker membrane.

Scanning Electron Microscope (SEM) is used to measure membrane thickness for *L* < 20 μm through the fabrication of a cross section layer using a Focused Ion Beam (FIB)^20^. Optical microscope is used to measure *L* of thicker membranes (*L* > 20 μm). In Fig. 3, representative SEM and optical images of some membranes are shown. For any sample, *L* is the mean value of repeated measurements in different points and the maximum error is the measurement error.

**Figure 4:**
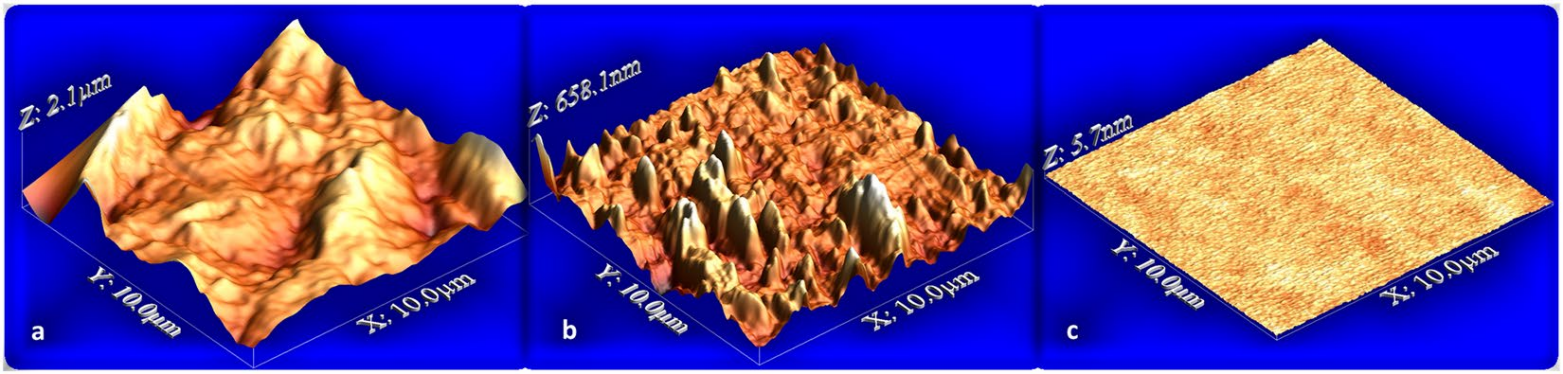
AFM images. (**a**) AFM 3D view of three membranes with *R*_*q*_ = 220 nm. (**b**) AFM 3D view membrane with *R*_*q*_ = 86 nm. (**c**) AFM 3D view of a flat surface *R*_*q*_ = 0.6 nm.

Atomic Force Microscope (AFM) monitors the surface morphology of flat and corrugated surfaces. By using WSxM freeware scanning probe software and averaging on several samples, we determined the flattened area A^*^ and the root mean square roughness of each membrane^21^. Figure 4 reports the AFM image of three membrane surfaces used in the present experiment. The root mean square roughness of the corrugated surfaces is *R*_*q*_ = 220 nm (Fig. 4a) and the ratio *ε* = A^*^/*A* between the flattened area and the surface area *A* is *ε* = 1.16 ± 0.05. The root mean square roughness of the corrugated surface of Fig. 4b is *R*_*q*_ = 86 nm and the ratio *ε* is 1.13 ± 0.05. The flat surface has *R*_*q*_ = 0.6 nm. All membranes used have been monitored with AFM, obtaining the same values of *R*_*q*_ within 10%. Table 1 reports *L*, *A* and *R*_*q*_ for all the membrane tested.

To check the goodness of the preparation procedure, Electron Dispersive X-Ray (EDX) has been carried out on rough surfaces to eventually reveal fluorine contaminants coming from the PTFE filter. Within the 1 μm of spatial resolution of EDX, the PDMS rough surfaces are not contaminated.

### Gas permeation apparatus

In order to measure the permeation rate *Q* through the membrane we have used an experimental apparatus, equipped with a Residual Gas Analyzer, based on selective on-line measurements of gas fluxes, reported in ref.^6^. Respect to the variable-volume or variable-pressure methods this set up allows to measure transient fluxes, directly, rapidly, and selectivity. To increase the accuracy of the measurements, respect to ref.^6^, the vacuum chamber has been equipped with a Spinning Rotor Gauge (SRG). *P*_*L*_ is determined by measuring the partial pressure difference across the membrane *Δp = p*_*u*_ − *p*_*d*_, where *p*_*u*_ is the upstream pressure and *p*_*d*_ the downstream pressure, and the gas flux *J* = *Q*/*A* as

6 $${{P}}_{{L}}=\frac{{Q}}{{A}{\rm{\Delta }}{p}}=\frac{{Q}}{A({{p}}_{{u}}-{{p}}_{{d}})}=\frac{{J}}{({{p}}_{{u}}-{{p}}_{{d}})}$$

The permeation rate *Q*, is the product of the pumping speed *s* of the system by *p*_*d*_. In this experiment, the SRG is used to calibrate the Quadrupole Mass Spectrometer (QMS), and *P*_*L*_ is measured at *p*_*u*_ = 1.013 × 10^5^ Pa. The error on *Q* depends on the uncertainty of *s* and *p*_*d*_, that, considering measurements of *p*_*d*_ after SRG calibration of QMS, has a relative error less than 5%. Taking into account the error on membrane area *A*, the relative error on *J* results less than 10%. Since the error on Δ*p* is less than 0.5%, the measured permeance has a relative error less than 10%. In the worst condition, the measurements of the membrane thickness *L* have an accuracy better than 20% as discussed in ref.^6^. The small roughness of the membranes does not affect the thickness value. The purity grade of the gases tested is N5.0.

## Results and Discussion

By following the procedure illustrated in section Method, we fabricated 5 FF, 6 FC and 5 CC membranes with different thickness with surface roughness *R*_*q*_ = 86 nm (see Table 1). FC have been measured in two different conditions: a) with the corrugated surface on the feed side and b) with the corrugated surface on the permeate side. The two configurations give the same permeance within the experimental errors. Gas permeation measurements for all the membranes are performed for two gases CO_2_ and N_2_.

**Figure 5:**
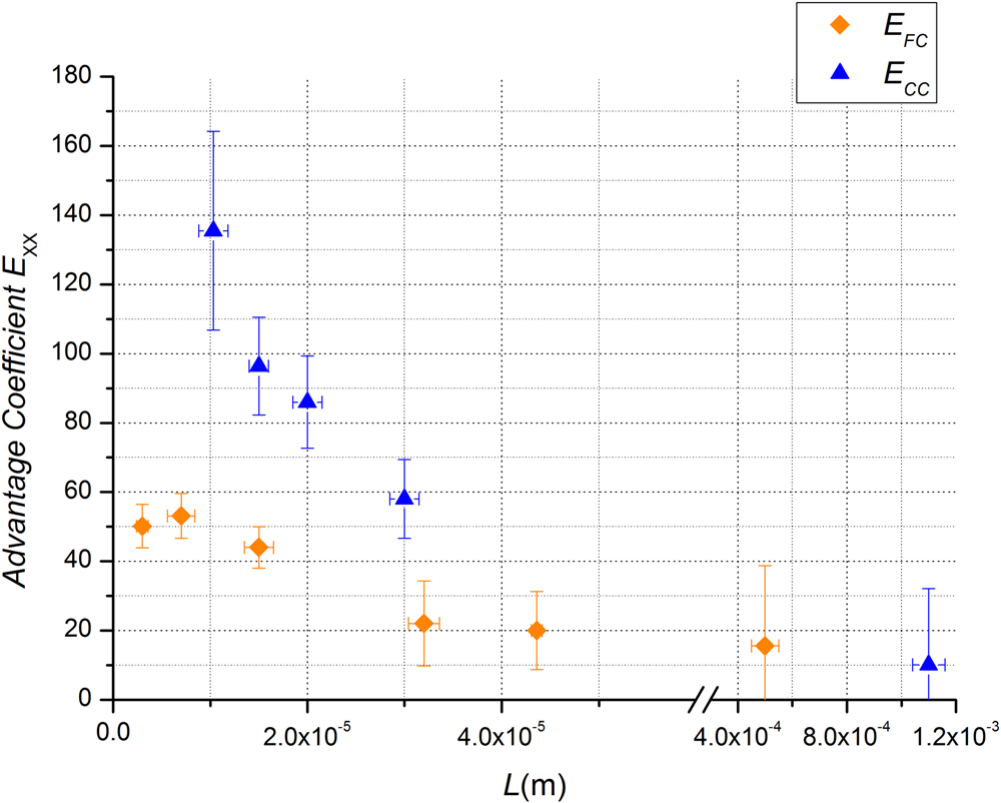
Advantage CO_2_ coefficient ${{E}}_{{XX}}$ as function of thickness ${L}$. ⬥ *E*_*FC*_, advantage coefficient for FC membranes ▲ *E*_*CC*_, advantage coefficient for CC membranes. The tracer gas is CO_2_, the upstream pressure is *p*_*u*_ = 1.013 × 10^5^ Pa and the temperature $T$ = 293 K.

Figure 5 reports the values of CO_2_ advantage coefficient *E*_*XX*_ defined in analogy to what reported in ref.^15^ as:

7 $${{E}}_{{XX}}=\frac{{{J}}_{{XX}}-{{J}}_{{FF}}}{{{J}}_{{FF}}}\times 100$$

**Figure 6:**
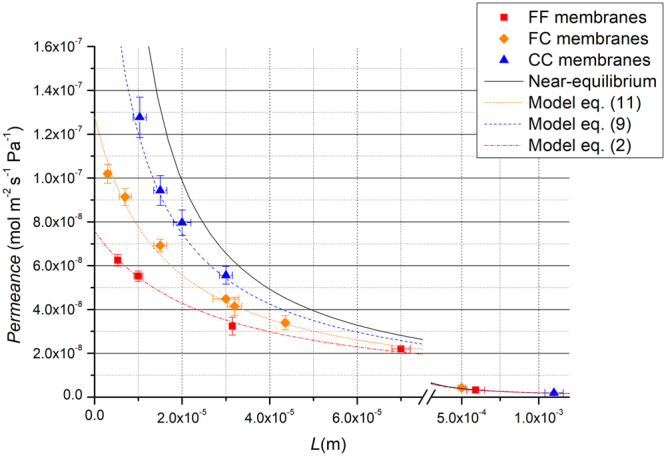
Permeance *P*_*L*_ of CO_2_ as function of thickness *L*. ◼ FF membranes ⬥ FC membranes ▲ CC membranes. The dotted lines are the best fit curves following the models of eqs (2), (8) and (10) respectively for FF, CC and FC membranes. The best fit parameters are reported in Table 2 for CO_2_. The continuous curve represents the condition of near equilibrium *P*_*L*_ = *P*_*HF*_/*L*. The tracer gas is CO_2_, the upstream pressure is *p*_*u*_ = 1.013 × 10^5^ Pa and the temperature $T$ = 293 K.

In eq. (7) *J*_*XX*_ is the flux, _*XX*_ indicates the specific membrane (FC or CC) and *J*_*FF*_ is the flux for the membranes with the flat surfaces. As shown in Table 1 the thickness of the flat membranes FF in most cases does not correspond to the respective thickness of the FC and CC membranes. The value of *J*_*FF*_ to calculate the advantage coefficient ${E}_{XX}$ defined by equation (6) is obtained by interpolating the curve that fits the permeance of the FF membranes reported in Fig. 6. The flux is always measured with the same *Δp* among the samples with different thicknesses, in particular with *p*_*u*_ = 1.013 × 10^5^ Pa and *p*_*d*_ < 10^−4^ Pa. *E*_*XX*_ quantifies the advantage obtained by using rough membranes respect to flat membranes.

The enhancement is significantly strong. FC membrane at *L* = 7 μm shows a permeance which is 53% larger than FF. The effect is even stronger when both surfaces are corrugated (CC membranes). At the smallest thickness (*L* = 10 μm) CC has an experimental advantage of 140% which corresponds to an increment in permeance of a factor 2.4 respect to the flat membrane and shows a permeance which is 61% of the value expected in near equilibrium conditions *P*_*L*_ = *P*_*HF*_/*L*. The effect is significant only at the micro-scale while at higher values of *L* the advantage coefficient is close to zero. In fact for CC at *L* = 1 mm, *E*_*CC*_ is 10% and for FC at *L* = 0,5 mm, *E*_*FC*_ is 15% but, due to the incertitude of the measurements, we cannot tell them apart from FF. Finally, we point out that the value of the permeability for FF (*L* = 0.5 mm) membranes is in agreement with that reported in the literature for the system CO_2_/PDMS of comparable thickness (*L* = 100 μm)^22^. The flux enhancement at low thicknesses cannot be explained by the increase in surface area due to the corrugation. In fact the value of $\varepsilon$ = 1.13 ± 0.05 measured by AFM is not able to justify the enhancement factor observed for CC and FC in the micro scale.

Figure 6 reports *P*_*L*_ as function of thickness *L* for FF, FC and CC membranes with surface roughness *R*_*q*_ = 86 nm for CO_2_. There is a strong enhancement of *P*_*L*_ at very low thickness, while, on the contrary, for macroscopic ones, *P*_*L*_ has the same values for FF, FC and CC. Since the characteristic length of the system is *L*_*C*_ = 30 µm, as recently determined^6^, the enhancement occurs in the SLR regime where the gas transport is surface sorption desorption rate limited. The rise is even more pronounced when both feed and permeate membrane surfaces are corrugated, CC has, in fact, a much larger permeance of both FF and FC.

The experiment shows that the surface resistivity *ρ*_*s*_ of permeate and feed surfaces decreases significantly respect to that of the flat surfaces. By defining the surface resistivity of the corrugated permeate and feed surfaces as *ρ*_*sc*_ = *βρ*_*s*_, where *β* < 1 the equations (4) and (2), valid for FF membranes, for CC and FC respectively assume the following form:

8 $${\rm{\Delta }}{p}=\frac{L}{{P}_{HF}}(1+\beta \frac{{L}_{C}}{L})\frac{{Q}}{{{\rm A}}}$$

9 $${{P}}_{{L}}=\frac{{{P}}_{{HF}}}{{L}+\beta {{L}}_{{C}}}$$

10 $${\rm{\Delta }}{p}=\frac{L}{{P}_{HF}}(1+(\frac{1+\beta }{2})\frac{{{L}}_{{C}}}{{L}})\frac{{Q}}{{{\rm A}}}$$

11 $${{P}}_{{L}}=\frac{{{P}}_{{HF}}}{{L}+(\frac{1+\beta }{2}){{L}}_{{C}}}$$

The analysis of the data has been performed first by fitting the permeance as function of thickness for FF membranes of Fig. 6 with eq. (2) by taking *P*_*HF*_ and *L*_*C*_ as fitting parameters. The results are reported in Table 2. Subsequently, for CC membranes, *P*_*L*_ is fitted with eq. (9) using *β* as fitting parameter and taking *P*_*HF*_ and *L*_*C*_ from Table 2. A similar procedure has been followed for FC membranes, *P*_*L*_ is fitted by using eq. (11) with *β* as fitting parameter, *P*_*HF*_ and *L*_*C*_ are taken from Table 2. The analysis is carried out with Least Absolute Residuals (LAR) method considering that the data have less anomalies and the coefficient of determination *R*^2^ describes the goodness of the fits^23^. We point out that the permeability measurements of FC membranes give the same values independently from which is the corrugated interface (up or downstream). The obtained values of *P*_*HF*_ and *L*_*C*_, listed in Table 2, are in agreement with those reported in refs^6,21^ and result more precise thanks to the improvement of the experimental set up (see section Method). The *β* values listed in Table 2 for CO_2_ gas are, within the experimental error, the same fitting the data with eq. (9) or with eq. (11) confirming the validity of the analysis.

**Table 2:** Permeability *P*_*HF*_, characteristic lengths *L*_*C*_, and parameter *β*.

	**CO**_**2**_				
	*P*_*HF*_ × 10^12^ (mol m^−1^ s^−1^ Pa^−1^)	*L*_*C*_ (µm)	*β* × 10^−1^	*β* × 10^−1^	*R* ^*2*^
FF	2.0 ± 0.4	26 ± 8			0.997
CC	2.0		2.2 ± 0.1		0.999
FC	2.0			2.2 ± 0.1	0.993
**N**_**2**_					
	***P***_***HF***_ **×** **10** ^**13**^ **(mol m** ^**−1**^ **s** ^**−1**^ **Pa** ^**−1**^ **)**	***L***_***C***_ **(µm)**	***β*** × **10**^**−2**^	*β* × 10^−2^	***R*** ^***2***^
FF	2.3 ± 0.4	14±4			0.998
CC	2.3		2.0 ± 0.4		0.999
FC	2.3			2.0±0.4	0.995
**He**					
	***P***_***HF***_ **×** **10** ^**13**^ **(mol m** ^**−1**^ **s** ^**−1**^ **Pa** ^**−1**^ **)**	***L***_***C***_ **(µm)**	***β*** × **10**^**−3**^		
FF	3 ± 1	13 ± 3			
FC			5 ± 2		
**CH**_**4**_					
	***P***_***HF***_ **×** **10** ^**13**^ **(mol m** ^**−1**^ **s** ^**−1**^ **Pa** ^**−1**^ **)**	***L***_***C***_ **(µm)**	***β*** × **10**^**−2**^		
FF	5 ± 1	17 ± 4			
FC			2.3±0.9		

**Figure 7:**
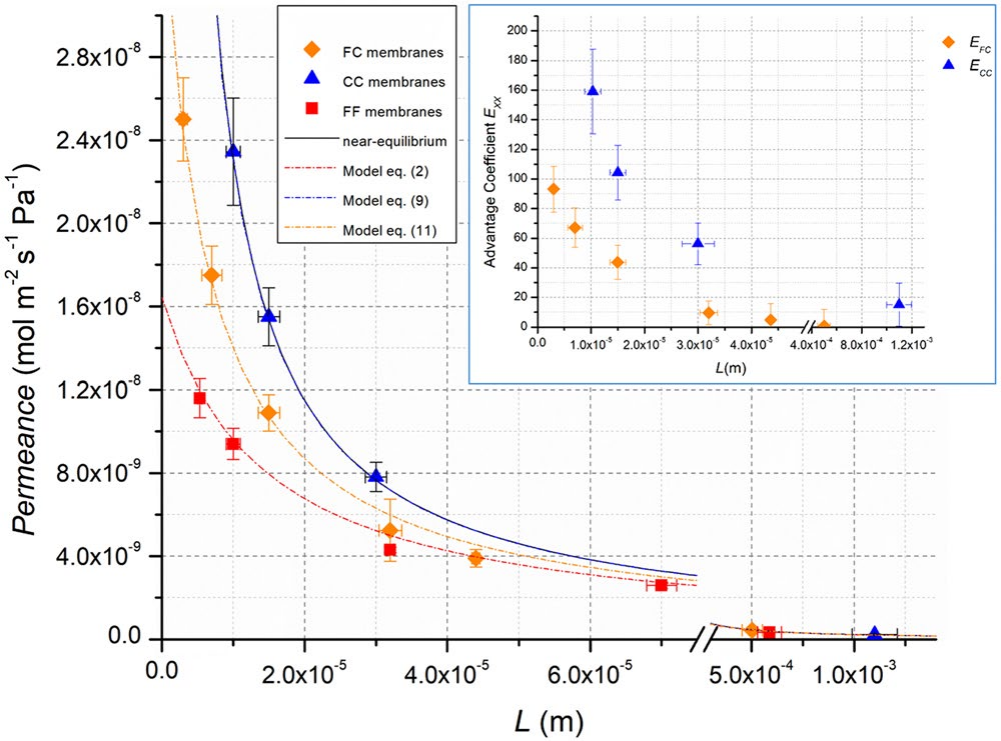
Permeance *P*_*L*_ and advantage coefficient ${{E}}_{{XX}}$ of N_2_ as function of thickness *L*. ■ FF membranes ◆ FC membranes ▲ CC membranes. The dotted lines are the best fit curves following the models of eqs (2), (8) and (10) respectively for FF, CC and FC membranes. The best fit parameters for N_2_ are those reported in Table 2. The continuous curve, that represents the condition of near equilibrium *P*_*L*_ = *P*_*HF*_*/L*, is overlapped on best fit curve of eq. (8). The tracer gas is N_2_, the upstream pressure is *p*_*u*_ = 1.013 × 10^5^ Pa and the temperature $T$ = 293 K. The inset shows the N_2_ advantage coefficient ${E}_{XX}$ as function of thickness $L$. ◆ *E*_*FC*_ for FC membranes ▲ *E*_*CC*_, for CC membranes.

In addition, we performed the same measurements by using N_2_ as tracer gas. Figure 7 reports *P*_*L*_ as function of thickness *L* for FF, FC and CC membranes with surface roughness *R*_*q*_ = 86 nm.

The trend of the permeance with thickness is very similar to that observed in the case of CO_2_. There is a strong enhancement of *P*_*L*_ at low thickness, that also in this case cannot be explained by an increase of the surface area. At large thickness, *P*_*L*_ assumes the same values for FF, FC and CC as occurs for CO_2_. The advantage coefficient in the inset shows that for this system the enhancement in permeance is even more pronounced. In this case, the model of equations (2), (9) and (11) describes quite well the observed behavior. The analysis of the data has been performed as previously described for CO_2_. Table 2 reports the values of the fitting parameters.

**Figure 8:**
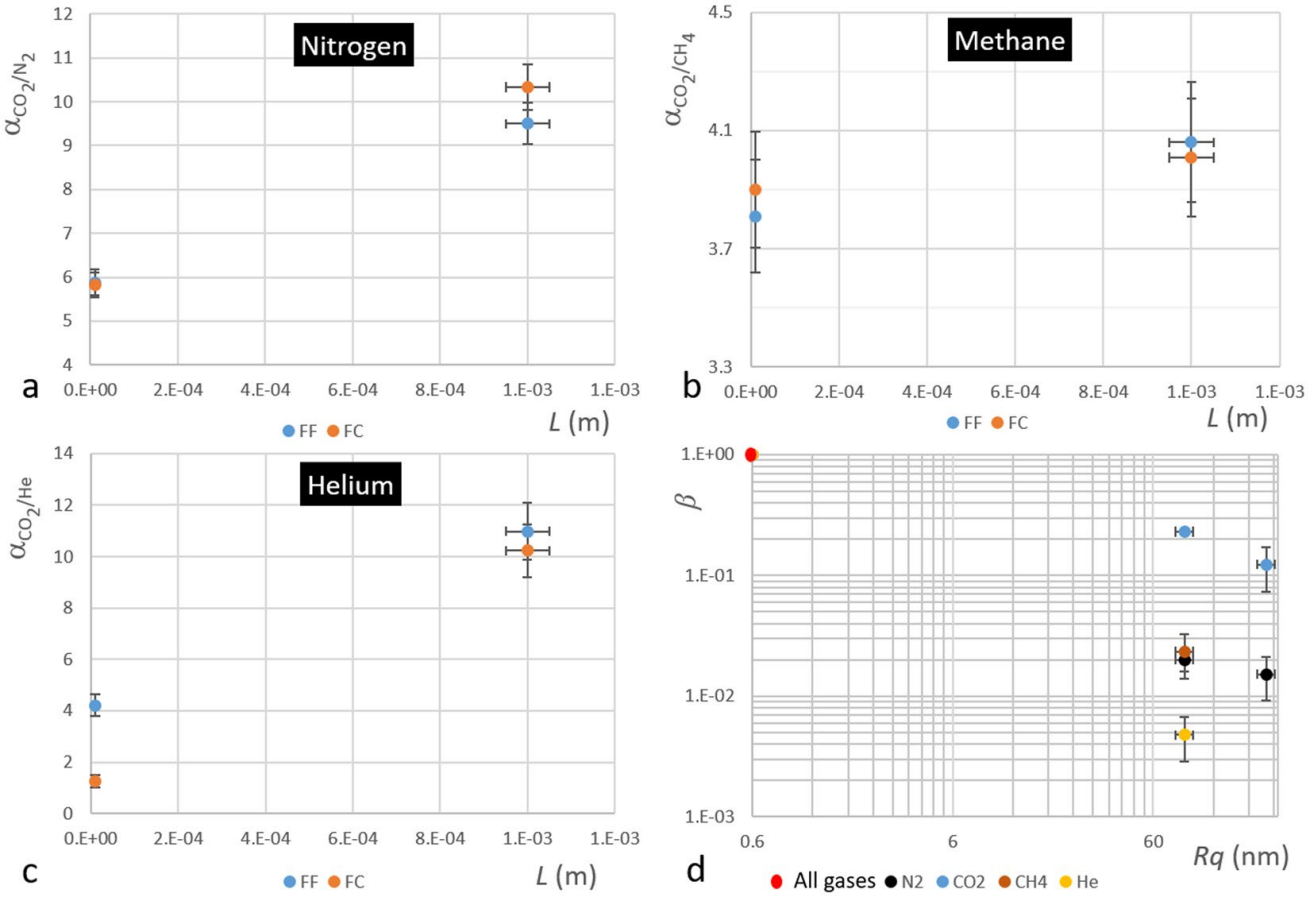
Selectivity as function of thickness L and surface resistance as function of corrugation *R*_*q*_. (**a**) CO_2_ selectivity respect to N_2_, (**b**) CO_2_ selectivity respect to CH_4_ and (**c**) CO_2_ selectivity respect to He for FF and FC membranes. FC membrane have the feed surface with a *R*_*q*_ = 86 nm. (**d**) Surface resistance for N_2_ CO_2_, CH_4_ and He at different surface roughness *R*_*q*_.

In addition, we measured the permeance of He and CH_4_ through two FF membranes with *L* = 2 mm and *L* = 10 µm and two FC membranes of the same thicknesses (data not shown). From these data, by using eq. (2) and performing the same analysis followed for N_2_ and CO_2_ we obtained the values of *P*_*HF*_, *L*_*c*_ and *β* reported in Table 2 for these gases. Figure 8(d) shows the parameter *β* for all four gases as function of roughness *Rq*. For CO_2_ and N_2_, an FC membrane with surface roughness *Rq* = 220, have been also tested and reported. It is clear from Fig. 8(d) that the change in resistivity depends on type of permeating gas.

In order to complete the analysis, we calculated from the permeability data the selectivity *α*_*ij*_ = *P*_*i*_/*P*_*j*_, where *P*_*i*_ is the permeability of the faster gas and *P*_*j*_ that of the slower gas, for CO_2_ respect to He, CH_4_ and N_2_. The results for FF and FC membranes are reported in Fig. 8(a,b and c).

The results for FF membranes follow the model of the permeability previously described^6^ since *L*_*C*_ of CO_2_ is grater then *L*_*C*_ for He, CH_4_ and N_2_ (see Table 2). The selectivity of CO_2_ respect to those gases decrease with *L* as shown in Fig. 8(a–c).

The results for FC membranes show that at *L* = 10 µm and *R*_*q*_ = 86 nm the selectivity coefficient *α*_*CO*2*/He*_ change significantly respect to those for FF membranes, while no change of *α*_*CO*2*/N2*_ and *α*_*CO2/CH4*_ occurs. Also in this case the effect is related to the modification of the surface resistance due to the roughness. These results suggest that, beside membrane permeance, the surface corrugation can modify also its selectivity. The change of both selectivity and permeance indicates that the effect is gas dependent.

## Conclusions

We fabricated PDMS membranes with nano-corrugated surfaces to study the effect of the corrugation on the permeation rate and selectivity. We measured CO_2_ and N_2_ permeance as function of membrane thickness *L* ranging from *L* = 3 µm to *L* = 1 mm. In both cases, we identified two different diffusion regimes (SLR and DLR) where the nano-corrugation plays a different role. In DLR, the permeation rate is practically independent of the roughness while in SLR the permeation rate increases significantly with respect to membranes with flat surfaces. The enhancement is particularly strong for CC membranes. For FC the effect is smaller and independent of which side of the membrane is rough. The flux enhancement at low thicknesses cannot be explained by the increase in surface area due to the corrugation. In the case of CO_2_ for the CC membranes with smallest thickness measured, *L* = 10 μm, the permeance increases by a factor of 2.4 with respect to the flat membranes. In this case the membrane shows a permeance which is 61% of the value expected in near equilibrium conditions *P*_*L*_ = *P*_*HF*_/*L*. When N_2_ is used instead of CO_2_, the effect is more pronounced.

The analysis of the data collected using both gases gives a satisfactory phenomenological explanation of the reduction of the surface resistance of the rough membrane with respect to the flat one. The same results are obtained in the case of He and CH_4_. The change of the surface resistance *R*_*S*_ with roughness affects also the selectivity of CO_2_ respect to He, CH_4_ and N_2_. The experimental results indicate clearly that the surface roughness influences significantly both membrane permeance and selectivity, offering an appealing method to increase the performance of thin membranes.

## Electronic supplementary material

Supplementary Information
