# Supplementary material for: Gas permeation through rubbery polymer nano-corrugated membranes
Source: Sci Rep. 2018 Apr 20;8:6345. doi: 10.1038/s41598-018-24551-4 (PMC5910414; doi:10.1038/s41598-018-24551-4)
Supplement: Supplementary file 1 — Supplementary Information [file 41598_2018_24551_MOESM1_ESM.pdf]

Gas permeation through rubbery polymer nano-corrugated membranes

Giuseppe Firpo *, Elena Angeli, Patrizia Guida, Roberto Lo Savio, Luca Repetto, Ugo Valbusa

Nanomed Labs, Physics Department, University of Genova, Via Dodecaneso, 33, 16146 Genova, Italy

* Corresponding author Email-address giuseppe.firpo@unige.it

Supplementary Information

The technique of fabrication of the PDMS membranes is critical to avoid artefacts. The first step is build PDMS membrane by spin coating with a rotational speed according to the desired thickness. The second step is transfer it on a silicon support as described in the membrane fabrication section, in the text. In the third step an identical silicon chip to that used for the support, called mask, is mounted on the PDMS and pressed against the polymer with a pressure of about 2×10^5 Pa (Fig. S1 a). Both silicon chips, the support and the mask, have an equal hole in the center, and the process of mounting the mask is such that the two holes are completely overlapped. The final result is a device with a PDMS freestanding membrane. The area exposed to the gas flow is the same from feed side and permeate side. With this procedure the PDMS layer is sandwiches between the two silicon chip, and thanks to the applied pressure on the mask, the surfaces of the polymer completely stick on the silicon surfaces.

At this point, the device is similar to that shown in Fig. 1 b. To install it in the experimental set up for permeation measurements, the device has been mounted on a perforated copper disk Conflat Flange© 16NW compatible. The leak tight adhesion on the copper disk has been guaranteed by means an epoxy resin Torr Seal© that stick together the device and the copper. A cross section of this assembly is shown in Supplementary figure S1.

The bonding between silicon and PDMS surfaces is such that there’s no partial bypass in a hypothetic marginal area between the noted area A and a less than perfectly adhered zone nominally covered by the

mask, even in case of rough surface. This fact is confirmed by two experimental results. First, FC have been measured in two different conditions: a) with the corrugated surface on the feed side and b) with the corrugated surface on the permeate side. The two configurations give the same permeance within the experimental errors. This means that the adhesion of corrugated and flat PDMS surfaces on the surface mask is the same.

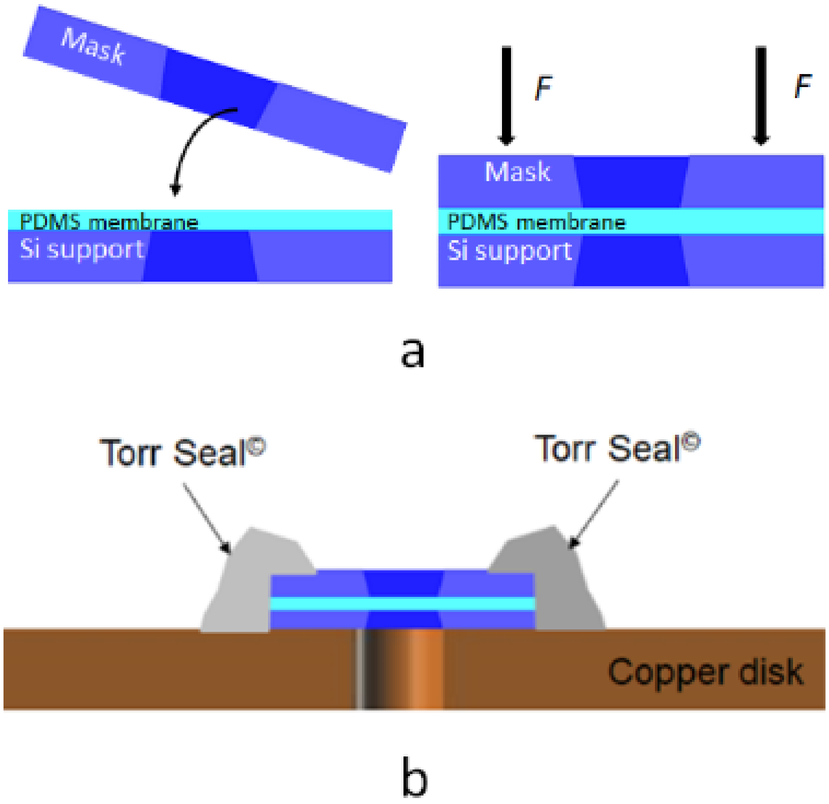

Fig. S1

Second, if the bonding between PDMS and the mask would not perfect, the gas would flow also through a potential “bypass area”. But, if this condition would real, the time to reach steady state would be longer than that measured. Actually, the thin membranes reach the steady state condition almost immediately after the application of 10^5 Pa on feed side. If the bypass area was present would have a very low conductance (at 86 nm the flow is molecular even at atmospheric pressure), the time for saturate the marginal area would be long and consequently also the response time of the system.

Figure legend

Fig. S1 Device fabrication and mounted on copper disk. (a) Installation of the mask on PDMS layer pressing it with a force *F* of about 12 N. (b) The device is placed in the center of the perforated copper disk, the PDMS freestanding membrane is overlapped on the hole of the copper disk. The Torr Seal© epoxy is the low vapor pressure resin that seal the device on the disk, guaranteed no measurable leak through the assembly
